# Optimized Clump Culture Methods for Adult Human Multipotent Neural Cells

**DOI:** 10.3390/ijms19113380

**Published:** 2018-10-29

**Authors:** Je Young Yeon, Ji-Yoon Hwang, Hye Won Lee, Hee-Jang Pyeon, Jeong-Seob Won, Yoo-Jung Noh, Hyun Nam, Kyeung Min Joo

**Affiliations:** 1 Department of Neurosurgery, Samsung Medical Center, Sungkyunkwan University School of Medicine, Seoul 06351, Korea; yeonjay.youn@samsung.com; 2 Stem Cell and Regenerative Medicine Center, Research Institute for Future Medicine, Samsung Medical Center, Seoul 06351, Korea; sandyky1020@gmail.com (J.-Y.H.); tadah881217@gmail.com (H.-J.P.); wjdtjq1124@gmail.com (J.-S.W.); nyj1350@gmail.com (Y.-J.N.); 3 Department of Anatomy & Cell Biology, Sungkyunkwan University School of Medicine, Suwon 16419, Korea; nsproper@naver.com; 4 Single Cell Network Research Center, Sungkyunkwan University School of Medicine, Suwon 16419, Korea; 5 Department of Health Sciences and Technology, SAIHST, Sungkyunkwan University, Seoul 06351, Korea

**Keywords:** adult human multipotent neural cells, clump culture, neural differentiation, angiogenic potential

## Abstract

Adult human multipotent neural cell (ahMNC) is a candidate for regeneration therapy for neurodegenerative diseases. Here, we developed a primary clump culture method for ahMNCs to increase the efficiency of isolation and in vitro expansion. The same amount of human temporal lobe (1 g) was partially digested and then filtered through strainers with various pore sizes, resulting in four types of clumps: Clump I > 100 µm, 70 µm < Clump II < 100 µm, 40 µm < Clump III < 70 µm, and Clump IV < 40 µm. At 3 and 6 days after culture, Clump II showed significantly higher number of colonies than the other Clumps. Moreover, ahMNCs derived from Clump II (ahMNCs-Clump II) showed stable proliferation, and shortened the time to first passage from 19 to 15 days, and the time to 1 × 10^9^ cells from 42 to 34 days compared with the previous single-cell method. ahMNCs-Clump II had neural differentiation and pro-angiogenic potentials, which are the characteristics of ahMNCs. In conclusion, the novel clump culture method for ahMNCs has significantly higher efficiency than previous techniques. Considering the small amount of available human brain tissue, the clump culture method would promote further clinical applications of ahMNCs.

## 1. Introduction

Neuronal death in human neurodegenerative diseases (NDs) is progressive and irreversible [1,2]. The lack of spontaneous recovery potential of the human adult brain makes it hard to reverse the disease process of NDs in the clinic [1,2]. Supplementation of exogenous stem cells might be a solution, since stem cells have innate potential to regenerate damaged tissues. Among various types of stem cells, mesenchymal stem cells (MSCs) are the most advanced in clinical trials for NDs [3]. However, MSCs may have limited regenerative potential for NDs due to their embryologic origin. Recently, neural stem cells (NSCs) have been introduced and focused on to treat NDs because of their superior neural differentiation potential [4,5,6,7].

NSCs reside in the specific regions of mammalian brains, including the subventricular zone and the subgranular zone [8,9,10]. NSCs are involved not only in the development of the central nervous system in the embryonic and fetal periods, but also in the regeneration of neural tissues throughout the lifetime. Recently, human NSCs have been recovered from the human fetal brain and cultured in vitro, exhibiting characteristics similar to NSCs from other mammalian species [11,12,13,14,15,16,17,18,19,20,21]. However, clinical use of human fetal NSCs harbors serious ethical issues, despite human fetal NSCs having been widely used in clinical trials for NDs, since there are few NSCs in human adult brains.

Previously, we introduced adult human multipotent neural cells (ahMNCs), NSC-like cells derived from the temporal lobe of adult epileptic patients [22]. In stroke and spinal cord injury animal models, significant therapeutic efficacies of ahMNCs have been demonstrated [22,23], suggesting their clinical implications without ethical problems. For clinical application of ahMNCs, it is important to acquire a sufficient number of cells. However, it is still difficult to isolate large numbers of ahMNCs because of the limited clinical availability of human adult brain tissue. In this study, we investigated highly efficient clump culture method to isolate and expand ahMNCs. Our data suggested that the novel clump culture method would upgrade the clinical utility of ahMNCs significantly.

## 2. Results

### 2.1. Experimental Design and Morphologies of Clumps

**Figure 1:**
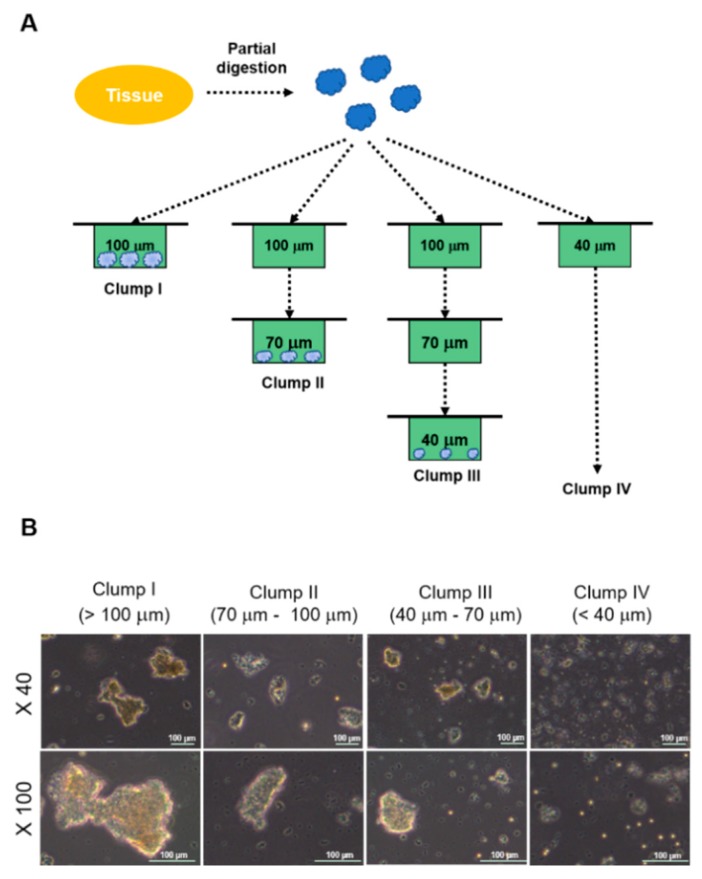
Experimental design and morphologies of clumps. (**A**) Experimental design to retrieve four types of clumps is illustrated. (**B**) Morphologies of four clump types is presented. Scale bar = 100 μm.

Human temporal lobe tissues (NS18-007TL and NS18-008TL) were divided into small pieces (1 g), and then partially dissociated into clumps physically and enzymatically. Each product was filtered through three kinds of cell strainers (100, 70, and 40 μm) in different combinations (Figure 1A). The filtration resulted in four types of clumps; >100 μm (Clump I), 70~100 μm (Clump II), 40–70 μm (Clump III), and <40 μm (Clump IV) from the same amount of surgical tissue (1 g, Figure 1A). The clump size of each clump group was validated by a microscope (Figure 1B).

### 2.2. Optimal Clump Size to Isolate and Expand ahMNCs

**Figure 2:**
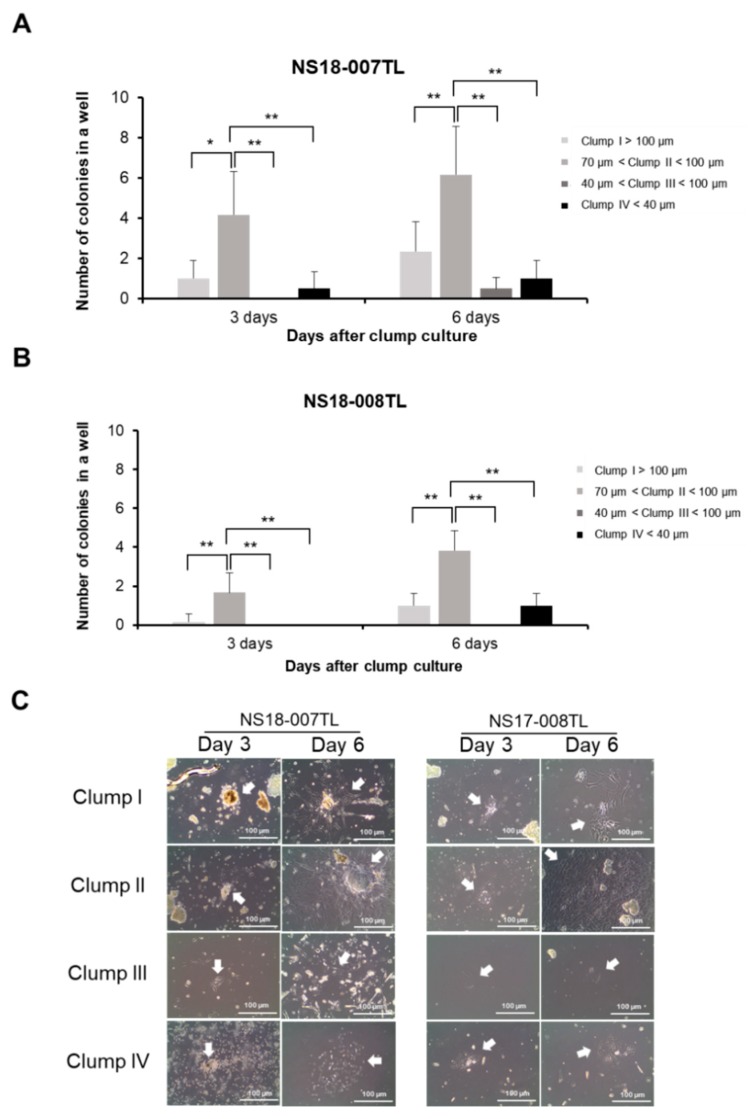
Colony forming efficiency of clumps. Adherent colonies from each clump-type were counted and photographed at 3 days and 6 days after in vitro culture ((**A**,**B**) respectively, *n* = 5 for each group). The number of adherent colonies from each clump-type was compared to each other ((**A**) for NS18-007TL and (**B**) for NS18-008TL). Height = average, error bar = standard deviation. * *p* < 0.05, ** *p* < 0.01. (**C**) Morphologies of adherent colonies are illustrated. Colonies are indicated by white arrows. Scale bar = 100 μm.

Each clump type was cultured under the conditions for ahMNCs [22]. At 3 days and 6 days after initiation of culture, morphology and number of colonies were determined under a microscope. At day 3, Clump II (70–100 μm) showed the highest number of adherent colonies (statistically significant, Figure 2A for NS18-007TL and Figure 2B for NS18-008TL). At day 6, the number of colonies was also highest in Clump II (statistically significant, Figure 2A for NS18-007TL and Figure 2B for NS18-008TL). The morphology of the clumps is illustrated in Figure 2C.

**Figure 3:**
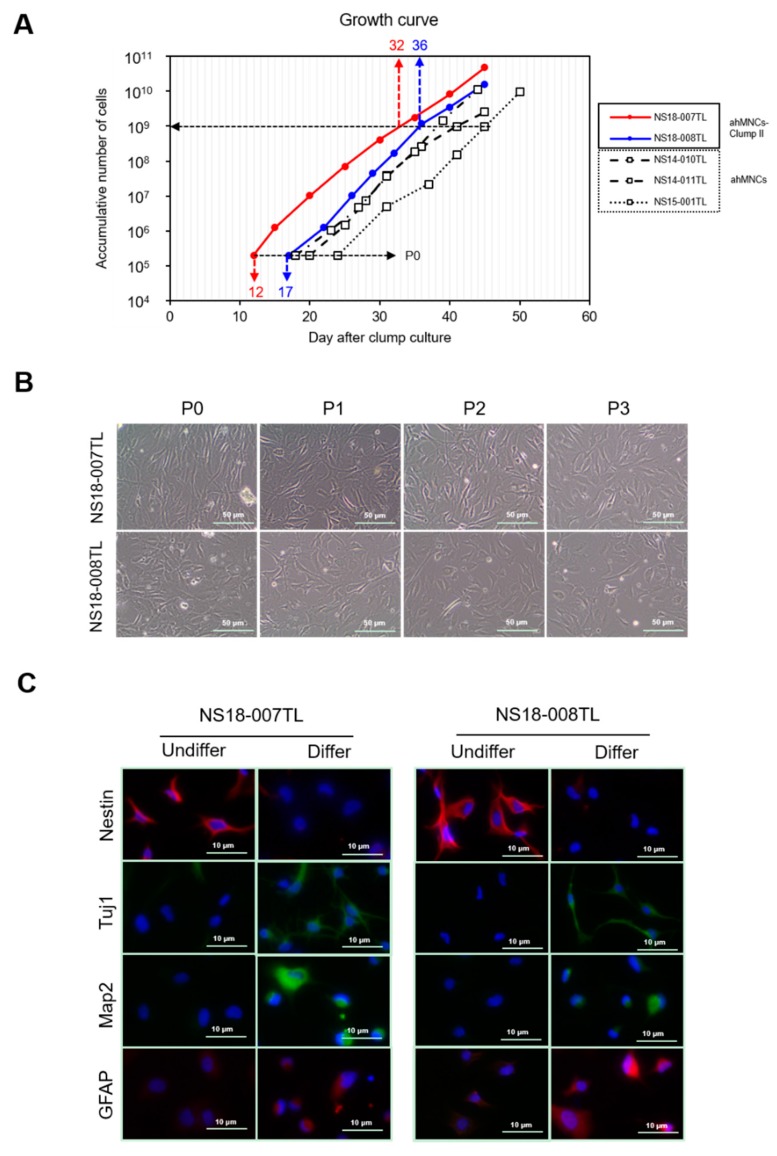
In vitro proliferation and differentiation of ahMNCs-Clump II. (**A**) ahMNCs-Clump II were propagated in the culture condition for ahMNCs. The accumulated number of cells of ahMNCs-Clump II (NS18-007TL and 008TL) in comparison with ahMNCs established using previous culture methods (NS14-010TL, NS14-011TL, and NS15-001TL). Days to 1 × 10^9^ cells are indicated. (**B**) Morphologies of ahMNCs-Clump II under expansion processes are illustrated until third in vitro passage (P3). Scale bar = 50 μm. (**C**) After in vitro differentiation, immunofluorescence was applied to ahMNCs-Clump II. Nestin for NSCs, MAP2 and Tuj1 for neurons, and GFAP for astrocytes. Undiffer = before in vitro differentiation; Differ = after in vitro differentiation. Scale bar = 10 μm.

Colonies from Clump II were future expanded in the adherent culture condition for ahMNCs [22]. Adherent cells derived from Clump II (ahMNCs-Clump II) were passaged serially in vitro and showed stable expansion (Figure 3A). The proliferation rates of ahMNCs-Clump II were comparable to those of ahMNCs (Figure 3A). However, the days of first passaging were 12 and 17 days for NS18-007TL and NS18-008TL, respectively, which were faster than those of ahMNCs using previous culture methods (18, 20, and 24 days for NS14-011TL, NS14-010TL, and NS15-001TL, respectively; Figure 3A). This shortened the production periods of ahMNCs (time to 1 × 10^9^ cells) from about 42 (39, 41, and 45 days, NS14-011TL, NS14-010TL, and NS15-001TL, respectively) to 34 (32 and 36 days, NS18-007TL and NS18-008TL, respectively) days (Figure 3A). The morphologies of ahMNCs-Clump II were like those of ahMNCs established previously [22] (Figure 3B).

### 2.3. Characterization of ahMNCs-Clump II

Characteristics of ahMNCs-Clump II were compared to ahMNCs established under previous culture methods [22]. The morphology of ahMNCs-Clump II was changed dramatically after the in vitro differentiation condition and showed dendrite-like branches. Immunofluorescence after differentiation showed a decrease in Nestin expression and an increase in the numbers of cells that were immunoreactive to Tuj1, MAP2, or GFAP (Figure 3C). With regard to the immunofluorescence, each marker was localized to the expected sites, showing specificity of the primary antibody. Tuj1- and Map2-positive dendritic processes and soma were prominent in differentiated ahMNCs (Figure 3C). However, there were few ODC-positive cells, which was in accordance with previous reports [22,23,24].

### 2.4. Angiogenic Potential of ahMNCs-Clump II

**Figure 4:**
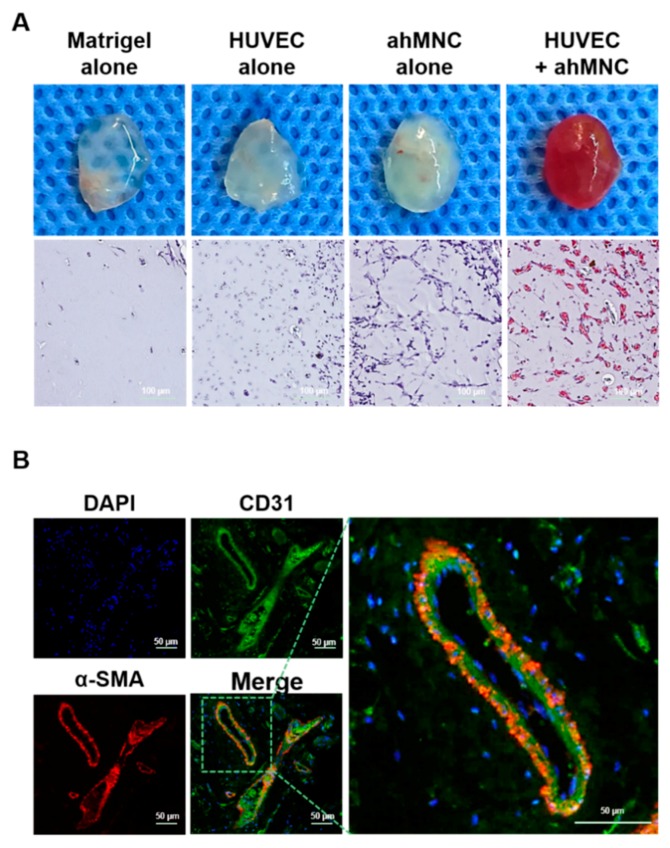
Angiogenic potential of ahMNCs-Clump II. (**A**) ahMNC-Clump II and/or HUVECs in Matrigel were injected into the subcutaneous tissue of immunodeficient mice. At 4 days after injection, Matrigels were retrieved and examined histologically. Scale bar = 100 μm. (**B**) Endothelial cells and pericytes were visualized by immunofluorescence for CD31 and αSMA, respectively. Scale bar = 50 μm.

Previously, we reported the angiogenic potential of ahMNCs [23]. The angiogenic potential of ahMNCs-Clump II was also determined in vivo by Matrigel plug assay. ahMNC-Clump alone and HUVEC alone made few microvessel-like structures with host-derived red blood cells (RBCs) (Figure 4A). In contrast, when ahMNCs-Clump II and HUVECs were co-injected, they resulted in highly vascularized Matrigel with host RBCs (Figure 4A). The microvessels were stained with CD31 and alpha-smooth muscle actin (α-SMA) to confirm the localization of endothelial cells and perivascular cells (Figure 4B).

## 3. Discussion

Multipotent NSCs were isolated and cultivated from various neurogenic regions of mature mammalian brains [19,25,26,27,28,29,30,31,32,33,34]. However, for clinical application, ex vivo expansion of NSCs is still crucial due to their rareness in adult brains. Conventionally, NSCs have been cultured by neurosphere culture methods [29,31,33,35,36,37,38,39,40,41]. We and others have reported that adherent culture methods showed better growth of NSCs compared to neurosphere culture methods, while cultured cells maintained their stemness and normal karyotypes [22,42,43,44]. Moreover, NSCs derived from younger human brains are more proliferative than those from older ones [45]. These results indicate the importance of microenvironment as well as the intrinsic characteristics of NSCs; a fair environment would guarantee the viability, maintenance, and proliferation of NSCs.

In this study, we introduced a clump culture method to isolate and expand ahMNCs from the temporal lobes of adult human brains. Previously, to establish a stem cell-enriched population, Percoll-based density gradient centrifugation was applied to dissociated cells [22]. Although blood cells, lipid, cell debris, etc., were efficiently removed by the Percoll-density gradient centrifugation, it also disconnects interactions between NSCs and microenvironmental cells. Based on the concepts of organoid culture [46], we hypothesized that the preserved microenvironment in clumps with appropriate size would promote the survival, stabilization, and proliferation of ahMNCs. In this study, the clump culture method demonstrated superior results to the conventional culture method [22].

In our clump culture method, the optimal size of clumps ranged between 70 µm and 100 µm (Clump II). Due to the difficulty in clump counting, all clumps derived from 1 g of tissue were plated without counting in this study. Considering that the colony numbers at day 3 and day 6 after seeding showed similar tendencies in two different experiments (NS18-007TL and NS18-008TL), the clump culture of this study might be reproducible. In Clump II, the adherent efficiency of partially dissociated cells was highest, which could result in the highest colony numbers at the first passage. This result is important for ex vivo expansion of stem cells. For clinical applications, shortening ex vivo expansion period would reduce production costs and increase success rate of primary isolation/culture. However, it is also necessary to validate whether cells derived from the clump culture method are true stem cells. In this study, the characteristics of ahMNCs-Clump II were determined via in vitro differentiation and in vivo angiogenic capacity. Like previous reports [22], ahMNCs-Clump II could differentiate into neurons (Tuj1^+^ and MAP2^+^) and astrocytes (GFAP^+^). In addition, in the previous article, we showed the electrical properties of ahMNCs after neuronal differentiation [22]. In accordance with previous reports [22,23,24], ahMNCs-Clump II showed limited differentiation potential into oligodendrocytes. This might be explained by the different characteristics of ahMNCs compared to fetal NSCs. In vivo Matrigel plug assay, ahMNCs-Clump II also showed similar angiogenic potential to the previous study [23].

In this study, we introduced a novel clump culture method for primary culture of ahMNCs and showed significantly higher efficiency than previous techniques. Considering the small amount of human brain tissue, our clump culture method with optimized clump size will be helpful for applying ahMNCs for various clinical purposes.

## 4. Materials and Methods

### 4.1. Study Approval

Informed written consents were obtained from each patient according to the guidelines approved by the Institutional Review Board of the Samsung Medical Center (SMC, Seoul, Korea) (IRB File No. SMC 2009-07-071 approved at 14 August 2009) to acquire surgical samples. Animal experiments were approved by the Institutional Animal Care and Use Committee (IACUC) of the Samsung Biomedical Research Institute (SBRI, Seoul, Korea) under approval number 20161019001 approved at 27 October 2016.

### 4.2. Clump Culture

Human temporal lobe tissue from two different patients was used in this study (NS18-007TL and NS18-008TL). The clinical information of the two patients (NS18-007TL and NS18-008TL) is provided in Table S1. Human temporal lobe tissue from adult epileptic patients was delivered in 4 °C saline and processed within 2 h after surgery. Surgical samples were weighed and divided into 1 g pieces. Each was separately minced and partially digested with enzyme solution containing 10 units/mL papain (Sigma, St. Louis, MO, USA), 0.1 mg/mL DNase I (Roche, Basel, Switzerland), and 4 mg/mL d, l-cysteine (Sigma) at 37 °C for 20 min. Clumps derived from each piece were filtered through cell strainers with three different pore sizes (40, 70 and 100 μm, Corning, NY, USA). After filtering, clumps were washed with PBS twice, and then cultured on poly l-ornithine (Sigma)-coated dishes in Dulbecco’s Modified Eagle Medium: Nutrient Mixture F-12 media (DMEM/F12) (Gibco, Paisley, UK) supplemented with 1× B27 supplement (Gibco, Grand Island, NY, USA), 1% penicillin/streptomycin cocktail (Gibco), 100 ng/mL epidermal growth factor (EGF) (R&D Systems, Minneapolis, MN, USA), 100 ng/mL basic fibroblast growth factor (bFGF) (R&D Systems), and 0.5% fetal bovine serum (FBS) (Gibco). Each type of clump was divided into 5 wells in a 6-well plate (Corning) and cultured. After 3 or 6 days, adherent colonies from each well were counted for statistical analysis and photographed. Cells were passaged further at 70% confluency.

### 4.3. In Vitro Differentiation

For differentiation, cells were cultured in DMEM/F12 supplemented with 1% penicillin/streptomycin cocktail, 0.5% FBS, 1× B27 Supplement, 100 ng/mL bFGF, 100 ng/mL EGF, and 0.5 mM 3-isobutyl-1-methylxanthine (IBMX) (Sigma) for 4 days, and then analyzed by immunocytochemistry.

### 4.4. Immunocytochemistry

Cells were fixed with ice-cold 4% paraformaldehyde (PFA) (Biosesang, Sungnam, Korea) for 10 min at room temperature (RT). Primary antibodies were treated overnight at 4 °C; anti-Nestin (1:500, NB100-1604, Novus, Saint Charles, MO, USA), Tuj1 (1:500, sc-5888, Santa Cruz Biotechnology, Inc., Dallas, TX, USA), MAP2 (1:250, sc-20172, Santa Cruz), and GFAP (1:1000, ab4674, Abcam, Cambridge, UK).

### 4.5. In Vivo Matrigel Plug Assay

Cells (total 2 × 10^6^) were suspended in 200 µL of ice-cold phenol red-free Matrigel (BD Bioscience, Franklin Lakes, NJ, USA) at ratios of 100:0, 50:50, and 0:100 (HUVECs:ahMNCs). The mixtures were then transplanted subcutaneously into the dorsal surface of Balb-c/nu mice (6-week-old, male) (Orient Bio, Seongnam, Korea) in accordance with previous reports [23,47,48]. At 4 days after injection, the Matrigel plugs were recovered, fixed with 10% buffered formalin, and then embedded in paraffin to prepare 4-µm-thick sections. For histological analysis, H&E staining was applied. For immunofluorescence, deparaffinized and rehydrated sections were incubated in target retrieval solution (Dako, Carpentaria, CA, USA) at 125 °C for 30 min. Anti-human CD31 (Santa Cruz Biotechnology) and anti-alpha smooth muscle actin (αSMA) (Dako) antibody were applied to slides at 4 °C overnight. Nuclei were counter-stained by DAPI (1:1000, ThermoFischer Scientific, Carlsbad, CA, USA) for 5 mins at RT.

### 4.6. Statistics

Data are presented as mean ± standard error. Data were analyzed by Student’s *t*-test, two-tailed. *p*-values < 0.05 were considered statistically significant.

## Supplementary Materials

Supplementary materials can be found at http://www.mdpi.com/1422-0067/19/11/3380/s1.
