# Supplementary material for: Optimized Clump Culture Methods for Adult Human Multipotent Neural Cells
Source: Int J Mol Sci. 2018 Oct 29;19(11):3380. doi: 10.3390/ijms19113380 (PMC6274905; doi:10.3390/ijms19113380)
Supplement: Supplementary file 1 [file ijms-19-03380-s001.pdf]

Supplementary Table 1. Clinical information of patients

Patients	**NS18-007TL**	**NS18-008TL**
Age at surgery	33	25
Sex	Female	Male
Seizure duration, years	16	12
Seizure frequency per months	1-2	4
Side of the brain	Left	Right
Perfusion on interictal SPECT	Mild hypoperfusion	Mild hypoperfusion
Hypometabolism on PET	Moderate hypometabolism	Mild hypometabolism
Intelligent quotient	81	80
Pathological diagnosis	Hippocampal sclerosis	Hippocampal sclerosis

* SPET: single photon emission tomography, PET: position emission tomography
